# Artificial Intelligence-Assisted Nanosensors for Clinical Diagnostics: Current Advances and Future Prospects

**DOI:** 10.3390/bios15100656

**Published:** 2025-10-01

**Authors:** Shuo Yin

**Affiliations:** Department of Biomedical Engineering, Stony Brook University, Stony Brook, NY 11794, USA; yinshuo0921@gmail.com

**Keywords:** nanosensors, biosensors, artificial intelligence, clinical diagnostics, disease biomarkers, point-of-care testing, precision medicine

## Abstract

The integration of artificial intelligence (AI) with various diagnostic nanosensors has opened up new horizons in clinics recently. AI technology offers enhanced sensitivity, accuracy, specificity, and real-time analysis for disease diagnostics. This review focuses on the recent advances in AI-assisted nanosensors for the diagnosis of different diseases in clinical applications. Critical roles of AI in sensor design, optimization, signal processing, and clinical decision support are highlighted. Furthermore, challenges such as limited datasets, regulatory hurdles, and data privacy are discussed, along with future opportunities. This review aims to provide a comprehensive introduction and perspectives on how AI-driven nanosensors are transforming clinical diagnostics and shaping the future of precise medicine.

## 1. Introduction

Nanosensors are biosensing devices that integrate different nanomaterials (NMs) with other sensing elements to detect biological or chemical targets [1]. By utilizing the unique physicochemical properties of NMs, such as a large surface-to-volume ratio, adjustable characteristics, high conductivity, and good biocompatibility, nanosensors offer enhanced performances over conventional biosensors. These include improved sensitivity, specificity, selectivity, faster response time, and the ability to perform multiplexed or real-time analysis in complex samples [2,3]. Nanosensors have found broad applications across healthcare, environmental and food monitoring, and industrial processes. In particular, their rapidly expanding role in healthcare highlights their significant potential in the clinical diagnostics, monitoring, and management [4,5,6]. Numerous nanosensors targeting disease biomarkers, including proteins, nucleic acids, and small molecules, have been developed to enable highly precise and non-invasive detections. Moreover, the emergence of microfluidics, point-of-care testing (POCT) devices, and wearable biosensors has further driven the advancement of personalized medicine [7,8,9,10].

Artificial intelligence (AI) refers to computational systems designed to perform tasks that typically require human intelligence, including perception, reasoning, decision-making, deep learning (DL), adaptation, and sensory processing [11]. In recent years, AI has been rapidly reshaping our daily lives and creating significant opportunities and challenges for the traditional healthcare system [12]. AI accelerates biomedical research and drug discovery [13,14], facilitates clinical screening and diagnostics [15,16], and supports with patient care. These applications help scientists, clinicians, and healthcare institutions deliver more accurate, efficient, and personalized care, highlighting the transformative role of AI in advancing modern healthcare and precise medicine [17]. 

Combining the strengths of nanosensors and AI technologies has led to the emergence of AI-assisted nanosensors, which provide smart, rapid, and highly sensitive tools for clinical diagnostics [18]. AI technologies contribute to nanosensor advancement by analyzing large datasets, identifying optimal recognition elements [19,20], and predicting sensor performance, thereby streamlining sensor design [21]. Moreover, AI can handle multidimensional and heterogeneous datasets for a more comprehensive evaluation of sensor parameters, accelerating the optimization of nanosensors with improved performances. Beyond sensor design and optimization, AI plays crucial roles in clinical diagnostics, including the analysis of data patterns, integration of various diagnostic indexes, real-time monitoring, and decision support, which further enhance the accuracy, efficiency, and clinical relevance of nanosensors [22]. Together, AI technologies have effectively promoted the broad use of nanosensors and driven their translation into clinical practice.

Some published reviews have introduced the applications of AI assisted nanosensors in healthcare. For example, Akkaş et al. [23] summarized various AI-assisted biosensors and discussed how AI can improve sensor performance. Taha et al. [24] focused on AI-assisted optical nanosensors and their applications in multi-omics research. Leong et al. [25] summarized machine learning (ML)-based design and fabrication of nanosensors and discussed their promising prospects in future pandemic outbreak. There are also reviews focusing on the application of AI-assisted nanosensors in specific types of diseases, such as cancer [26], viral diseases [27], and bacterial infections [28]. Based on the above, this review will systematically summarize the application of AI-assisted nanosensors in the diagnosis of various clinical diseases (cancers, infectious diseases, cardiovascular diseases, metabolic disorders, etc.), highlight the multi-scenario applications of AI technologies in sensor design, fabrication, and clinical cases, and also discuss the limitations and future directions of AI-assisted nanosensors in clinical diagnostics.

## 2. AI-Assisted Nanosensor Design and Clinical Application 

**Figure 1:**
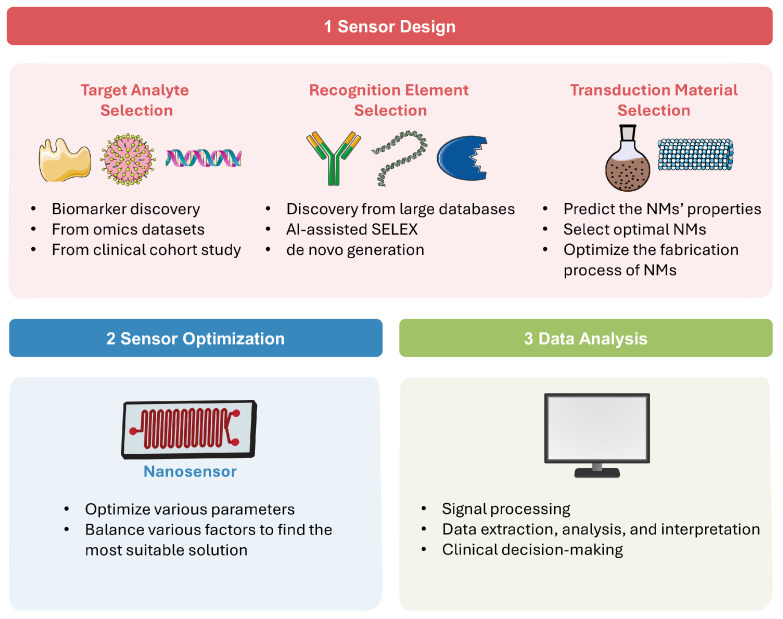
AI-assisted workflow in sensor design, optimization, and data analysis.

AI technologies have become a powerful tool for developing and utilizing various nanosensors [29]. In this section, we will explore different AI technologies in detail, discuss how they are integrated across nanosensor design and application, and highlight their principles and advantages. A schematic overview of the AI-assisted workflow in nanosensor is shown in Figure 1.

### 2.1. AI in Sensor Design 

As present in Figure 1(1), a diagnostic nanosensor typically consists of three main components: the analyte, which is usually a disease-specific biomarker; the recognition element, which selectively interacts with the analyte; and the transduction material, which converts this interaction into a readable signal, such as optical, fluorescent, colorimetric, electrochemical, and thermal outputs [30]. The following paragraphs explore the AI applications across different sensor design components.

#### 2.1.1. Target Analyte Selection

Biomarkers are biological molecules that are confirmed to be associated with specific diseases, and their concentrations are often correlated with disease severity, progression, and clinical outcomes [31]. Biomarkers can take various forms. For example, in certain viral and bacterial infections, the pathogens themselves may serve as biomarkers. In cancer, biomarkers include proteins, nucleic acids, circulating tumor cells (CTCs), exosomes, and others [32]. The selection of an appropriate target analyte is critical to the nanosensor performance, influencing the sensitivity and accuracy for disease diagnostics. In recent years, AI technologies have been widely employed to analyze high-throughput omics data/clinical data and to identify novel biomarkers with greater efficiency and precision [33]. Mass spectrometry (MS)-based proteomics generates complex and multi-dimensional data. AI algorithms trained on these omics’ datasets can uncover disease-associated protein expression patterns. By applying appropriate classification models, these patterns can be correlated with healthy/diseased states, thereby enabling high-throughput screening of potential disease-specific biomarkers [34]. For example, Deng et al. [35] developed an explainable DL method to analyze MS data from 859 serum samples and screen potential biomarkers for lung cancer. The detection accuracy for early-stage lung adenocarcinoma reached 96.1%, with an area under the curve (AUC) of 0.99. Hällqvist et al. [36] designed a ML classifier trained on MS-based plasma proteomics data for the early screen of Parkinson’s disease. An eight-protein panel (GRN, MASP2, HSPA5, PTGDS, ICAM1, C3, DKK3, SERPING1) was identified to distinguish Parkinson’s patients with 100% accuracy. In addition, AI technologies have also been used to discover potential biomarkers from clinical cohort study data and electronic health record data. For instance, Ge et al. [37] developed a ML-based prognostic biomarker screening method using pancreatic adenocarcinomas cohort study data. Biomarkers and potential therapeutic targets, such as PLEC, TRPV1 and ITGB4, were identified. Mataraso et al. [38] established a multimodal ML approach based on electronic health records and transfer learning to improve the analysis of small omics datasets, achieving more precise patient identification and biomarker discovery. It is worth mentioning that the accuracy of AI model screening depends on sufficient and high-quality training set data. The variability of training data quality across various sources will affect the accuracy and reliability of AI screening results [39]. Furthermore, before clinical transformation, biological experiments are also required to verify the physiological and biological relevance of these candidates.

#### 2.1.2. Recognition Element Selection

The recognition element serves as the foundation of specific detection for a nanosensor. Therefore, ideal recognition elements require strong and selective affinity toward the target analytes [40]. They are commonly categorized into natural elements, such as antibodies (Abs) and enzymes, which rely on natural physiological interactions, and synthetic elements which are artificially engineered to achieve specific binding, including aptamers, peptides, nucleic acids, G-quadruplexes, and molecularly imprinted polymers (MIPs) [41]. AI technologies can help with the selection of sensor recognition elements by analyzing known molecular and binding data to predict the physicochemical properties of candidates, and by modeling unknown or complex interactions to identify novel recognition elements [42]. One major application is the AI-assisted Ab discovery from large databases. Traditional experimental discovery is often labor-intensive and time-consuming; thus, AI technologies are employed to automate and accelerate key stages of the Ab discovery process, including selection, characterization, and developability maturation [43]. For instance, da Silva et al. [44] developed a new ML method, epitope3D, for accurate epitope prediction. It models epitope and non-epitope regions as graph-based structural signatures, capturing spatial patterns to train ML models. Following selection, affinity evaluation lies at the core of Ab characterization step. Recently, a growing number of AI models have been introduced for Ab affinity evaluation [45]. In the final stage, some random forest (RF) algorithms, neural networks, and pre-trained language models (LMs) are also applied to predict and optimize the developability of Abs [46,47]. Another important application is ML-powered systematic evolution of ligands by exponential enrichment (SELEX) techniques for aptamer discovery. For example, Song et al. [48] employed multilevel structural analysis and unsupervised ML to develop a sequential multidimensional algorithm for highly efficient, accurate, and robust aptamer discovery. Di Gioacchino et al. [49] applied an unsupervised neural network model, restricted Boltzmann machines (RBMs), to learn from SELEX data, enabling accurate prediction and generation of high-affinity aptamers. Similarly, ML algorithms have also been used for MIP design [20] and peptide discovery [50]. A third area where AI plays a key role is in the de novo generation of Abs. Some deep generative modeling techniques are employed to design and optimize Ab sequences, such as long short-term memory (LSTM) networks [51], variational autoencoders (VAEs) [52], generative adversarial networks (GANs) [53] and LMs [54]. For example, Hie et al. [54] performed Ab affinity maturation with general protein language models (GPLMs) and greatly improved binding affinities of Abs across only two laboratory evolutions. Without relying on target-specific information, GPLMs enable efficient affinity maturation by proposing plausible Ab mutations. Overall, these AI technologies can accelerate the nanosensor development process by enabling rapid and efficient discovery of optimal recognition elements. It is especially valuable for responding to emerging infectious diseases caused by novel pathogens.

#### 2.1.3. Transduction Material Selection 

In nanosensors, transduction materials are NMs which can convert molecular recognition reactions between the analyte and the recognition element into detectable signals. Various NMs have been applied in nanosensors, including metallic NMs, carbon-based NMs, magnetic NMs, and various composite-based NMs [1]. It plays a critical role in determining the signal transduction efficiency and sensitivity of the nanosensor. AI models can predict the NMs properties based on large datasets, select the optimal materials for sensitive and selectivity sensing detection, and optimize the fabrication conditions of NMs [55,56]. For example, Shiba et al. [57] analyzed sensing data with Gaussian process regression (GPR) and selected the most suitable functionalized nanoparticles for enhanced accuracy of sensor detection. Malakar et al. [58] employed LSTM and feedforward neural networks (FFNN) to accurately predict the mechanical properties of monolayer transition-metal dichalcogenides under varied conditions based on data from molecular dynamics simulations. This approach is also expected to predict optical, electrical, and magnetic properties of NMs, bringing it closer to practical applications in sensor design. Xu et al. [59] applied backpropagation artificial neural network optimized to assist the fabrication of a nanosensor based on multi-walled carbon nanotubes (MWCNTs)/graphene oxide (GO)/dendritic silver nanoparticles (AgNPs) nanohybrid and to achieve intelligent sensing of target analytes. These examples highlight the great potential of AI technologies in supporting the selection of transduction material and advancing the development of advanced nanosensor devices.

### 2.2. Sensor Optimization

Following the design of the three fundamental elements of the nanosensor, it is crucial to optimize various parameters to obtain best detection performance. In real-world applications, factors such as sensitivity, cost, accuracy, and detection efficiency must be balanced to find the most suitable solution. As summarized in Figure 1(2), AI technologies can balance various sensor parameters, find the optimal solution, and thus significantly accelerate the optimization process while improving its accuracy. Wekalao et al. [60] designed a tunable terahertz metasurface nanosensor for malaria diagnosis. The XGBoost ML algorithm was applied to predict and optimize sensor performance across various design parameters, achieving peak performance in detecting minute changes in parasite concentration. Patel et al. [61] proposed a sensor behavior prediction method based on polynomial regression to analyze three types of metasurface designs, effectively optimizing sensor sensitivity through design and parameter variation. Jemmali et al. [62] introduced a hybrid model combining particle swarm optimization (PSO) algorithm and artificial neural networks (ANN) to accurately predict biosensor detection times. This model demonstrated strong predictive capabilities and represents a promising tool for advancing biosensor technology. Bajahzar et al. [63] optimized the PSO-ANN model using the Box–Behnken design and applied it to enhance the performance of a lab-on-disk biosensor, resulting in significant improvements (R^2^: 99.9%). This work involved complex multiphysics simulations and careful AI model optimization, demonstrating innovation in overcoming computational challenges in sensor design and optimization. Appadurai et al. [64] employed a polynomial regression-based ML model to assist in the design of the proposed plasmonic sensor, achieving high prediction accuracies ranging from 92% to 100%. This ML model effectively captured complex nonlinear relationships in sensor responses and offered a reliable and efficient predictive tool for optimizing. 

### 2.3. Data Analysis and Clinical Decision-Making

Raw sensor signals are frequently affected by noise, baseline drift, or nonlinear behaviors, which may hinder accurate data interpretation. AI technologies offer powerful tools to process complex sensor signals, can adapt learning across diverse sensor systems and signals [29]. Aalizadeh et al. [65] integrates multi-resonance nanostructures with ridge regression ML model to achieve highly precise parameter prediction of biosensor. This method obtained up to three orders of magnitude improvement in accuracy without altering the sensor hardware. Zhang et al. [66] employed dynamic signal change analysis combined with a recurrent neural network (RNN) to enhance biosensor accuracy (98.5%) and detection speed. This strategy effectively reduces false positives and shortens response delays for the quantification of miRNA (let-7a) across a broad concentration range from nM to fM. Esmaeili et al. [67] applied short-time Fourier transform (STFT) to preprocess electrochemical sensor datasets obtained from the detections of 35-mer adenosine, 31-mer oestradiol and 35-mer oestradiol, and subsequently used various DL models to identify the optimal approach for accurate data classification and analysis. This work highlights the potential of DL models in processing complex time-series signal processing tasks.

Beyond signal processing, AI technologies can extract data patterns and perform data classification, providing an accurate and efficient way to make smart clinical decisions [33]. Using AI models, different analytes can be simultaneously discriminated and quantified [68,69]. Vaiyapuri [70] tested the improvement performances of four ML models on infectious disease diagnostic models, used Shapley values to reasonably interpret the models, and revealed various clinical features that affect diagnosis, thereby supporting physicians in making clinical decisions. Alam et al. [71] presented a shuffle shepherd optimization-based generalized deep convolutional fuzzy network (SSO-GDCFN) strategy to analyze COVID-19 diagnostic sensor data and identify disease states, types, and rocovered categories. This method demonstrated excellent diagnostic performance, reaching 99.99% accuracy within minimal reclassification process. Liu et al. [72] integrated nanophotonic biosensor and DL model for the ultrasensitive detection of encephaloduroarteriosynangiosis biomarker S100B. The DL model ProSpect was developed for automated image analysis, enabling rapid, matrix-flexible, and ultrasensitive detection with a LOD of 1 pg/mL.

Sensor data is often complex and unstable, while disease diagnosis involves diverse biomarkers, mixed data sources, and various sample types. AI technologies can help process sensor data better and faster, explore small changes and hidden features in the data, achieve accurate data analysis and interpretation, thereby supporting clinical decision-making and ultimately improving AI-driven healthcare systems (Figure 1(3)). These examples mentioned above is just a very small fraction of AI applications in this field. More examples for specific disease diagnostics will be discussed in detail in Section 3.

## 3. AI-Assisted Nanosensors for Disease Diagnostics

AI technologies enhance nanosensor design and optimization while also supporting complex data analysis for clinical decision-making. However, in clinical practice, detecting different diseases requires distinct diagnostic strategies. For instance, infectious disease requires rapid and accurate detection to prevent spreading, whereas cancer diagnostics typically rely on multi-biomarker profiling and advanced classification models with high sensitivity for early screening. Cardiovascular disease normally benefits from warning system and long-term monitoring. Similarly, chronic diseases, such as metabolic and neurological disorders, require monitoring and feedback functions to support disease management. To address these clinical needs, various AI models have been developed integrated with nanosensors. In this section, we summarize and discuss the recent clinical applications in AI-assisted nanosensors in the diagnosis of specific diseases, including infectious diseases, cancers, cardiovascular diseases, metabolic disorders, and neurological diseases (Table 1).

### 3.1. Infectious Diseases

In recent decades, numerous emerging and re-emerging infectious diseases have threatened public health. Notable examples of infectious diseases include severe acute respiratory coronavirus (SARS-CoV) in 2003, the Middle East respiratory syndrome coronavirus (MERS-CoV) in 2012, the Ebola virus epidemic from 2013 to 2016, Zika virus outbreak in 2015–2016, and SARS-CoV-2 emerged in 2019 and spread worldwide. Meanwhile, some long-standing infectious diseases, such as acquired immunodeficiency syndrome (AIDS) and tuberculosis, have continued to bring heavy burdens on public health [108]. Infectious diseases are caused by live pathogens and are capable of rapid transmission and infection among humans. Therefore, early, accurate, and rapid diagnostic techniques are crucial for effective disease management [109]. To address these challenges, various nanosensors have been developed to improve the speed and sensitivity of pathogen detection compared to traditional techniques such as polymerase chain reaction (PCR) and enzyme-linked immunosorbent assay (ELISA) [110]. The integration of nanosensors and AI technologies offers a promising approach to further improve the accuracy and efficiency of infectious disease diagnostics [111].

**Figure 2:**
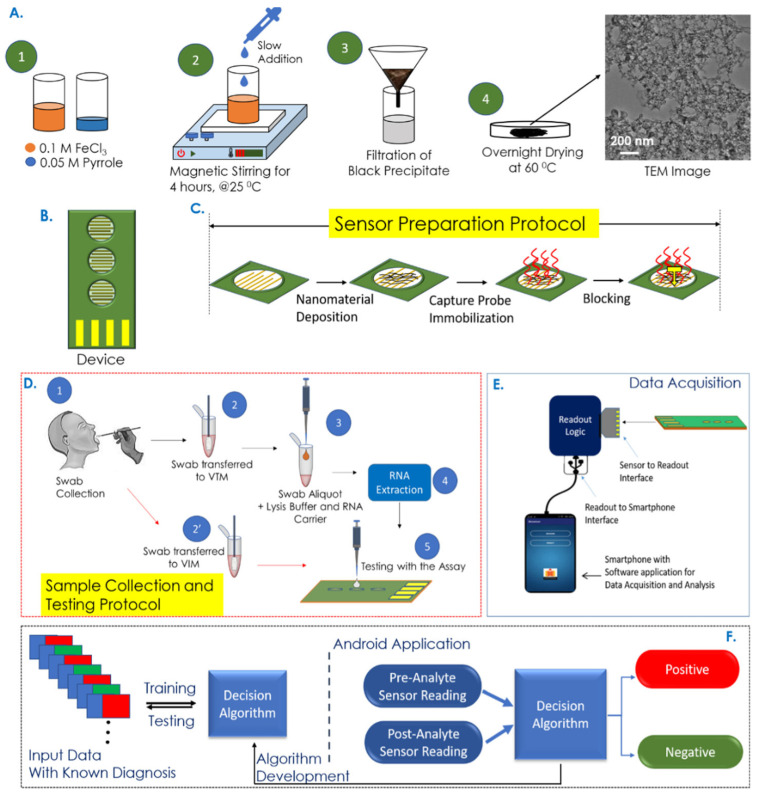
Overview of the COVID-19 rapid detection process utilizing a chemiresistive biochip platform integrated with mobile application/AI model assisted data analysis. Reproduced from Ref. [74]. Copyright 2021 American Chemical Society.

Electrochemical sensors have metrics such as improved sensitivity, lower limit of detection (LOD), and faster response time, opening new possibilities for early and rapid disease diagnostics [112]. Garcia-Junior et al. [73] selected bio-inspired peptide using AI and developed a peptide-based electrochemical sensor for the detection of SERS-CoV-2 in human saliva sample. The peptide probe can recognize the receptor binding domain (RBD) of the viral spike protein and generate electrochemical signal changes. A support vector machine (SVM)-based ML model was used to analyze electrochemical data and improve the detection sensitivity and specificity. Sukjee et al. [81] developed a MIP-based electrochemical sensor for the detection of Zika virus in urine. Variations in urine protein and glucose can affect the sensor’s signal trends by Zika virus concentrations. To address this, a SVM model was introduced to unify varying signal trends, reduce noise, and thereby improve the reliability and accuracy of virus detection. While electrochemical biosensors remain widely used due to their high sensitivity, chemiresistive biochips have gained increasing attention as an alternative electrical detection strategy. For example, Tripathy et al. [74] established a rapid COVID-19 testing platform based on a nucleotide probe-functionalized chemiresistive biochip. As shown in Figure 2, the capture probe was immobilized on the polypyrrole NM-based biochip, enabling specific hybridization with viral RNA from swab samples. The resulting resistance changes were collected by a portable readout device connected to an Android application. A binary separable dataset was generated using a SVM classifier for training/testing. By comparing different supervised-learning classifiers, the support vector classifier with a radial basis function (SVC/RBF) achieved the highest accuracy of 95.2% for extracted RNA samples and 100% for non-extracted samples. 

Moreover, AI-assisted optical biosensors have also been used for viral detection. Vishalatchi et al. [75] developed a two-dimensional photonic crystal-based optical sensor for the detection of sexually transmitted viruses. A k-Nearest Neighbors (kNN) ML model was employed to train and test the sensor data and calculate the accuracy. The proposed optical sensor achieved an accuracy of 97.12% and high sensitivity at 998 nm. Zhao et al. [76] developed a negative/positive discrimination method based on surface-enhanced Raman scattering (SERS) scanning imaging and a ResNet-18 DL model to analyze SERS sensor data for rapid virus detection. The DL-assisted SERS sensor simplifies the detection process, reduces the need for specialized personnel and detection time, and enhances diagnostic reliability. Beisenova et al. [80] developed a multiplexed nanoplasmonic biosensor for profiling COVID-19 immunity. RF model was applied to evaluate and classify the immunity status across populations based on responses to six SARS-CoV-2 antigens. This ML model significantly streamlined the data analysis process and provided a powerful tool for serological diagnostics.

Besides applying single nanosensors, AI technologies have been employed to analyze big datasets and optimize prediction models. The Internet of Things (IoT) is composed of various devices that can sense and communicate with each other. IoT generates large volumes of biomedical data and plays a vital role in automatic medical data collection systems and large-scale disease prediction models [113]. Maheshwari et al. [114] employed a fuzzy-based decision tree (FDT) algorithm to classify data from nanotechnology-based IoT biosensors. Satisfactory performances were achieved when analyzing unstructured datasets and incomplete medical records. This method provides an improved way to assist physicians in predicting infectious diseases and supporting clinical decision-making.

### 3.2. Cancers

Cancer continues to be a leading cause of death globally and presents a significant barrier to improvements in life expectancy [115]. Early diagnosis of cancer is crucial for improving prognosis and increasing survival rate [116]. Compared with conventional methods, biosensors offer highly sensitive, specific, and non-invasive detection of cancer biomarkers, with great potential for integration into precision and personalized medicine. Due to the complexity and heterogeneity of cancer, accurate diagnostics often rely on the detection of multiple biomarkers [117]. Various AI technologies have been developed and employed to analyze sensor data in cancer diagnostics, extract meaningful information, and enhance the sensor overall performance and diagnostic reliability [118].

A variety of cancer biomarkers can serve as targets for sensors, supporting non-invasive diagnosis of cancer. Carcinoembryonic antigen (CEA) is a common tumor biomarker associated with several cancers. Lei et al. [88] developed a microwave biosensor for the detection of CEA in human serum. A convolutional neural network (CNN) model was applied to predict and validate the concentrations of the target and further support the simulation modeling. The CNN model improves prediction accuracy by automatically extracting and learning nonlinear feature patterns from the response data, capturing key variations in the curves while reducing dimensionality, which preserves essential information and enhances the model’s ability to generalize. The diagnostic accuracy was verified by Western blot, and consistent results were obtained. Tumrani et al. [89] established a gold-decorated Ti_3_C_2_T_x_/porous carbon-based electrochemical immunosensor the detection of breast cancer biomarker extracellular matrix protein-1 (ECM1). An ANN model was trained to capture the nonlinear relationship between sensor responses and analyte concentrations and was applied to the predictive analysis of differential pulse voltammetry data, thereby validating its practical applicability in cancer diagnostics. Redín et al. [90] applied feature selection to analyze voltammograms obtained from a protein p53 immunosensor. Different ML techniques, including logistic regression, linear discriminant analysis, kNN, Gaussian Naive-Bayes, decision trees, and SVM, were used to enhance the sensor’s performance. The results showed that even a sensor with initially limited capabilities can be greatly improved through applying suitable features and AI models, highlighting the potential of AI technologies in diagnostic sensors. 

Some biosensors have been used to detect cancer cells/tissues and to further classify cancer samples. Pathak et al. [83] used an ANN model to analyze data generated by a 2D-photonic crystal biosensor. Based on the confusion matrix, distinct differences in the behavior of electric flux and refractive index were observed among various cancer cell types. This strategy demonstrated improved predictive performances for cancers, achieving 81% accuracy for skin cancer and 89% sensitivity for blood cancer. Balaji et al. [85] proposed a 2D photonic crystal biosensor for the detection of various cancer cells and applied multiple linear regression (MLR) and SVM to repeatedly train, test and optimize the sensor data, allowing real-time analysis and intelligent interpretation. SVM enhances predictive capability by mapping multidimensional response signals into a higher-dimensional space and constructing an optimal hyperplane to accurately model the relationship between sensor features and analyte concentrations. The results indicated that the SVM demonstrated better performance with a R^2^ of 0.99, enhancing the accuracy and sensitivity of the biosensor. Wekalao et al. [86] designed a metasurface surface plasmon resonance (SPR) biosensor for cervical cancer diagnostics. Support vector regression (SVR) with a polynomial kernel was applied to analyze the sensor data and obtained an optimal R^2^ of 100%. This AI model is less sensitive to outliers and can be optimized through parameter tuning, thereby generating an optimal regression function that minimizes prediction error. Taking the advantages of AI model, the proposed sensor can distinguish normal and cancerous cervical tissues based on refractive index differences, showing its potential as a non-invasive and accurate tool for cervical cancer diagnosis. 

**Figure 3:**
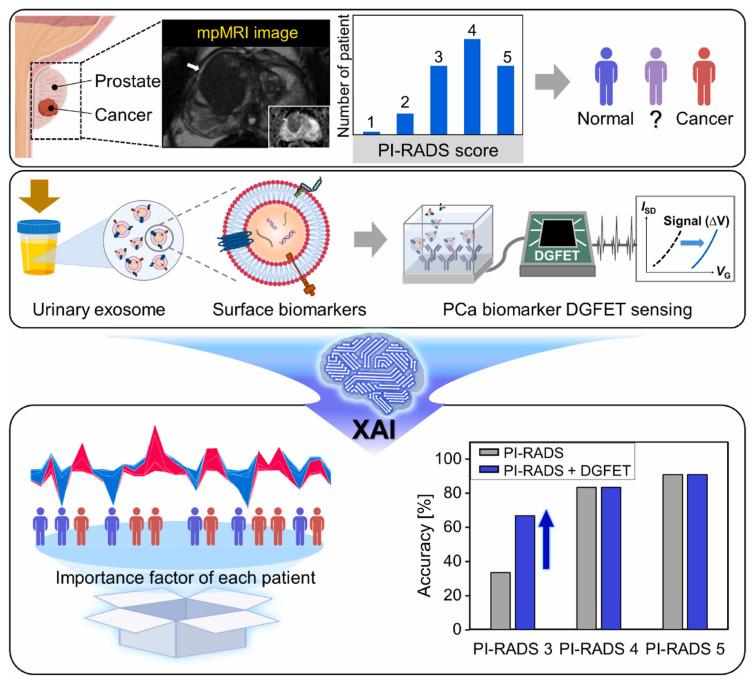
Schematic illustration of XAI-based DGFET biosensor for the prostate cancer screening. Reproduced from Ref. [84]. Copyright 2024 Elsevier.

Simultaneous detection of multiple biomarkers can enhance the diagnostic accuracy of cancers and is widely used in cancer diagnostics. Wang et al. [82] designed a lateral flow chip sensor based on gold nanoparticles (GNPs) and fluorescent nanodiamonds (FNDs) composites for the simultaneous detection of miRNA-21 and miRNA-96, both recognized as early biomarkers of breast cancer. To enhance signal interpretation, data preprocessing and signal convolution techniques were applied to both the chromatographic signals from the test line and the fluorescence spectra of the nanocomposites. SVR and GPR were utilized to predict miRNA concentrations with high accuracy. This technique enables rapid detection within 5 min, achieving fM level LOD and an R^2^ value of 0.9916. Xu et al. [91] developed an electrical sensor for the simultaneous detection of miRNA-451 and miRNA-145. Various models were employed to predict miRNA detection, incorporating data collection, feature extraction, model training, and validation. Among them, the RF model showed the best performance and significantly enhanced the sensitivity and accuracy of cancer diagnostics. Choi et al. [84] developed a novel prostate cancer screening method by integrating a dual-gate field-effect transistor (DGFET) biosensor with explainable artificial intelligence (XAI) (Figure 3). Three exsomal biomarkers, TMEM256, flotillin-2, and PSMA, were detected and used for XAI analysis. For patients with a prostate imaging reporting and data system (PI-RADS) score of 3, this method achieved twice the diagnostic accuracy compared to traditional assessment. Unlike black-box AI models, the XAI technique is interpretable and transparent, providing insights into the significance of visualized multi-biomarkers and clinical factors. Thus, it assists physicians in delivering more accurate and evidence-based prostate cancer diagnostics. Albano et al. [87] applied 34 types of ML classifiers to analyze electrochemical sensor data for establishing an oral squamous cell carcinoma diagnostic model. The resulting AdaBoost AI model integrated multiple biomarkers and achieved superior accuracy of 76%. Apart from detecting specific biomarkers, disease diagnostic can also be achieved by classifying profiles from different samples. For example, Sunil et al. [92] applied SVM to classify the SERS profiles from healthy controls and lung cancer patients. A 94% classification accuracy was obtained, which is highly valuable for lung cancer diagnostics.

The above examples of AI-assisted biosensors for cancer diagnostics show that applying AI technologies can make up for the limitations of a sensor’s initial performance, improve detection sensitivity, and enhance the capture and processing of data features. Together, these advances increase classification accuracy and support more precise and earlier cancer diagnosis.

### 3.3. Cardiovascular Diseases

Cardiovascular diseases are one of the leading death causes worldwide, contributing to substantial morbidity, reduced quality of life, and a significant economic burden on society. Moreover, population aging is expected to further increase the prevalence of many cardiovascular diseases in the near future [119]. Most cardiovascular diseases are acute and require rapid diagnosis followed by prompt treatment. Accurate diagnosis of cardiovascular diseases often relies on monitoring the dynamic trends of specific biomarkers or indicators. Therefore, continuous monitoring can improve early detection and diagnostic accuracy [120]. Various nanosensors and wearable biosensors have been developed for the diagnosis and monitoring of cardiovascular diseases and AI technologies have been employed to analyze sensor data, thereby enhancing sensitivity and accuracy [121]. In addition to traditional sensors, wearable biosensors provide a non-invasive diagnostic approach and have advanced the development of precision medicine in recent years. They can continuously monitor the user’s physiological signals and issue warnings when abnormalities occur, help with early screening and disease diagnosis, monitor and manage chronic diseases to achieve personalized and precise medicine [122]. However, wearable devices rely more heavily on intelligent data analysis and interpretation to ensure accuracy. To improve reliability and accuracy, AI technologies have been used for data extraction, analysis, and interpretation of their output data of wearable devices [123]. Chen et al. [97] developed a highly sensitive SERS wearable sensor for detecting cholesterol in sweat and employed a RF model to classify samples from cardiovascular patients and healthy people. An accuracy of 83.5% was obtained, showing its potential for non-invasive cardiovascular disease diagnosis. Jain et al. [98] applied a ML model with a LightGBM classifier to classify and evaluate the cardiac conditions based on data collected from wearable devices. LightGBM uses a leaf-wise tree growth strategy to improve performance and can handle high-dimensional data with categorical features. The AI model achieved an accuracy of 94.49% on the test set, with precision, recall, and F1-scores all above 0.95. Some data from wearable devices is incomplete and lack precision and consistency due to varying usage environments. Appropriate AI models can help users and physicians overcome these data limitations to achieve more accurate predictions. Talaat et al. [124] proposed a hybrid AI model, CardioRiskNet, that performs data preprocess, data feature selection, encoding, XAI integration, active learning, evaluation, and validation. Taking advantage of AI and active learning, this model overcomes data limitations and provides a powerful tool for assessing and predicting cardiovascular disease risk. 

**Figure 4:**
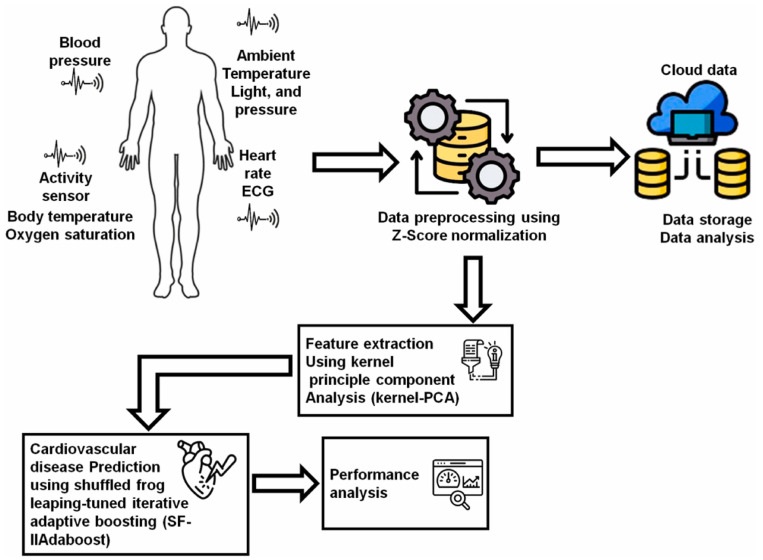
The flow of the AI-IoT-driven cardiovascular disease prediction model. Reproduced from Ref. [93]. Copyright 2024 Elsevier.

IoT technologies can monitor user’s health indicators, issue alerts when detecting abnormalities, and support remote patient care. AI technologies participate in analyzing data from various wearable sensor devices and establishing diagnostic models based on IoT platforms [125]. Marengo et al. [93] used Kernel principal component analysis to extract data features from medical sensors and wearable devices and obtained a dataset to train a Shuffled Frog leaping-tuned iterative improved adaptive boosting algorithm for predicting cardiovascular diseases (Figure 4). The proposed model achieved satisfactory performance, with an accuracy of 95.37%, a precision of 93.51%, and a sensitivity of 94.3%. Yashudas et al. [94] developed an IoT-based remote cardiovascular disease prediction system, DEEP-CARDIO, to predict, diagnose, and classify cardiovascular diseases. In addition to achieving high accuracy of 99.90%, this system can also provide users with physical and dietary suggestions via a mobile application. Islam et al. [95] developed a cardiovascular disease risk-level prediction system based on wearable sensors and IoT technology. A ML model with stacking classifier was employed to predict users’ disease levels, including a two-level system (presence/absence of disease) and a three-level system (low/moderate/high risk), offering a promising remote strategy for real-time disease monitoring and risk management. Kanza et al. [96] proposed a fuzzy logic and ML model based early detection solution and trained various ML models with Cleveland heart disease dataset. The results proved that RF had the highest accuracy of 82.6%, providing a new method for early diagnosis of cardiovascular diseases.

### 3.4. Metabolic Disorders

**Figure 5:**
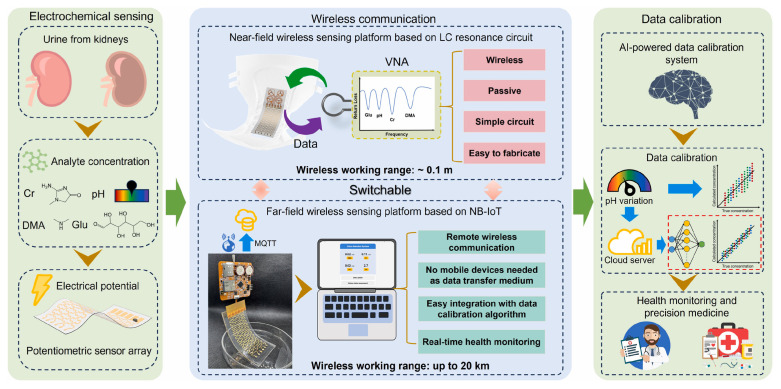
Principle and procedures of the MLP-assisted wearable device for detecting biomarkers in urine. Reproduced from Ref. [99]. Copyright 2025 Elsevier.

Similarly to cardiovascular diseases, the diagnosis of metabolic disorders often benefits from the multiplex biomarker detection and the long-term monitoring of indicator trends, which together enable early diagnosis and support effective disease management [126]. Diabetes is generally considered the most prevalent metabolic disease worldwide and requires routine glucose monitoring to help maintain and improve quality of life. Kumar et al. [100] introduced a 3D-printed electrochemiluminescence biosensor for the detection of glucose and lactate. ML algorithm was used to improve the accuracy of predicting biomarkers of diabetes. Chellamani et al. [101] designed a non-invasive glucose monitoring method based on photoplethysmography. Federated learning was proposed to replace the deep neural network strategy that relies on data sharing among different healthcare institutions. This method enables multiple institutions to collaboratively train an AI model without sharing data, thereby ensuring data privacy and security. Satisfactory accuracy was achieved with a root mean square error of 19.1 mg/dL. Gragnaniello et al. [102] applied a 1D-CNN and spectrogram preprocessing method-based Edge-AI technique to analyze ECG data from wearable devices and assess the presence of diabetes. This method achieved an accuracy of 89.52%, proving its potential for diabetes monitoring in resource-constrained environments. Chenani et al. [103] developed a bilayer hydrogel-based wearable patch for sweat monitoring of sweat pH and glucose levels and employed RF and CNN for data analysis. The intergrated AI model enhanced the consistency and accuracy of the wearable sensor across different devices and environmental conditions. Dong et al. [99] developed a wireless and wearable sensor integrated in diaper for detecting four urine biomarkers, including dimethylamine, creatinine, glucose, and H^+^ (Figure 5). Multilayer perceptron (MLP)-based data auto-calibration technology was employed to correct the errors caused by different pH values in urine samples and thus enhance the detection accuracy.

### 3.5. Neurological Disorders

Some neurological disorders, such as Alzheimer’s disease and Parkinson’s disease, are described as a silent pandemic due to limited non-invasive diagnostic methods and effective treatments [127]. To address the challenges of achieving non-invasive, accurate, and reliable diagnosis from limited yet diverse data collected by biosensors and wearable devices, AI technologies have been increasingly employed. Clinical samples usually contain various interferents which influence the accuracy of detection. Xu et al. [106] applied ML algorithms to assist in detecting and distinguishing multiple aggregates of Aβ40 and Aβ42. This approach obtained 100% accuracy in identifying aggregate species and demonstrated the applicability to diagnose Alzheimer’s disease. Kim et al. [105] developed a SERS biosensor in combination with a DL model to assist in analyzing Alzheimer’s disease. The DL model automatically extracted informative features and classified SERS spectra from healthy controls and patients. Accuracies of 96%-100% were achieved, highlighting its potential for the diagnosis of Alzheimer’s disease. Kavungal et al. [104] developed a nanoplasmonic infrared metasurface sensor for detecting proteins associated with neurodegenerative disorders. A deep neural network (DNN) model was integrated with the sensor to quantitatively predict the oligomeric and fibrillar protein aggregates within mixed samples. The proposed method allows the generation of structural fingerprint maps of diverse protein biomarkers and provides valuable information for disease diagnostics, monitoring, and therapy. Wang et al. [107] employed ML models to analyze and integrate various plasma biomarkers of Alzheimer’s disease and discriminate different stages of disease progression. Compared with traditional techniques, this method provides superior selectivity and sensitivity (LOD: fM level) for detecting disease-related biomarkers, achieving an AUC of 0.94 for accurate diagnosis of disease progression.

## 4. Real-World Applications of AI-Assisted Nanosensors

**Figure 6:**
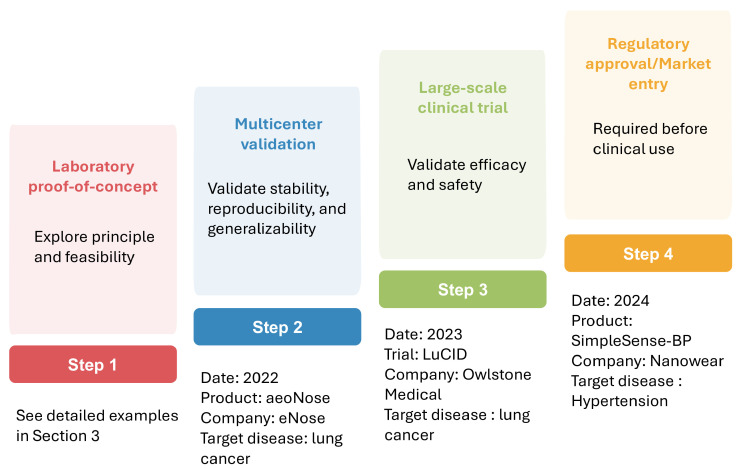
Translation of AI-assisted sensors from lab to clinical application.

Numerous studies have demonstrated the promise of AI-assisted nanosensors in improving clinical diagnostics. However, most of these advances remain in laboratory settings, with only a very limited number having progressed into real-world clinical applications. Figure 6 illustrates the steps involved in the clinical translation of AI-assisted sensors from laboratory to real-world applications, along with representative examples at each stage. Multicenter validation evaluates method stability, reproducibility, and generalizability across diverse institutions, populations, and environments, is among the first critical steps in translating research findings into real-world applications [128]. For example, the eNose company has completed a multicenter validation study evaluating the aeoNose device, which is based on a volatile organic compound (VOC) nanosensor combined with an ANN model, for lung cancer detection. The study included 575 subjects and achieved a sensitivity of 95%, a specificity of 49%, and a negative predictive value of 94% [129]. Following the confirmation of reliability in multicenter studies, large-scale clinical trials are conducted to validate efficacy and safety. A representative example is the LuCID study by Owlstone Medical company. This study employed VOC sensors coupled with ML models for early lung cancer screening. It recruited 4000 participants from 26 sites in the U.K. and Europe and investigated the diagnostic accuracy in this large-scale population. However, the results indicated that these breath biomarkers showed limited diagnostic potential for early lung cancer detection in the relevant clinical population [130]. Upon completion of large-scale trials, regulatory review is required before the method can be implemented in routine clinical practice. Notably, Nanowear’s SimpleSense-BP is currently the first and only U.S. Food and Drug Administration-cleared AI-assisted wearable nanosensor. This device provides real-time, clinical-grade cardiovascular data for continuous blood pressure monitoring and can assist clinicians in initial classification [131]. This example highlights the promising potential of AI nanosensors for translation into real-world clinical practice.

## 5. Challenges and Future Directions 

AI-assisted nanosensors have enormous potential in clinical diagnostics, especially in precision medicine. The previous section provided examples of AI’s practical applications in diagnostic sensors, but it is important to note that this field still faces several challenges. First, there are issues related to the data itself. The signals collected by sensors are influenced by noise, environmental factors, and individual differences, resulting in data of relatively low quality. Some new disease biomarkers or models have limited datasets, which are often single-source and imbalanced, making it difficult to train robust AI models. Additionally, selecting and training outstanding AI models is challenging depending on the application scenario. For example, some black-box models lack data explainability, interpretability, and traceable biological evidence, which may reduce clinicians’ trust; some AI models may overfit, performing well in laboratory but failing to meet expected performance in new cohorts/hospitals; for wearable devices, they require better computational power and real-time capabilities of AI algorithms. Second, there are challenges in clinical translation. AI algorithms lack standardized and unified evaluation systems, necessitating clinical trials between actual applications, which consume time and cost. From a technical perspective, clinical translation requires seamless integration of AI into standard workflows with user-friendly interfaces and compatibility with existing management systems. Additionally, doctors and patients are highly sensitive to misdiagnosis and have limited trust in new AI models. Thirdly, there are ethical and privacy concerns. Medical data is sensitive information, and the use in AI model training may lead to data breaches. During AI model training, the limited scope of the participating population may also generate medical ethical challenges. 

To address these challenges, the future directions can be explored through several key areas. First, AI can accelerate the design process of nanosensors and participate in sensor optimization, including surface modification and NM/probe design. AI algorithms can also accurately predict sensor performance based on existing data, thereby reducing the workload and costs associated with wet lab experiments. It is particularly important for emerging infectious diseases, as the rapid development of diagnostic tools is crucial for avoiding disease spread. Furthermore, some wearable sensors may require multiple detection mechanisms, such as electrochemical and optical signals. AI algorithms can be employed for signal fusion calculations and decision-making processes. 

The second part involves AI participating in the data analysis of nanosensors to achieve intelligent analysis. For example, AI models can analyze sensor data from different sources to improve diagnostic accuracy; AI can learn from small sample datasets and perform transfer learning to address new disease models. Early diagnosis relies on sensors with sufficient sensitivity, but sensor data often contains a lot of background and interference signals. Through AI models, these signals can be denoised to extract useful information, achieving sensitive detection. Some Explainable AI models are also suitable for diagnostic sensors, as they can help physicians understand the reasons/logics for AI’s decision-making, which is beneficial for clinical application. Disease diagnosis requires multidimensional information, and future efforts should focus on AI methods for handling multimodal and asynchronous data for clinically interpretable multimodal diagnostics.

The third part mainly focuses on AI-supported personalized medical care. For example, AI can assist real-time monitoring and issue warnings when abnormalities are detected based on wearable devices. It can also monitor and analyze patient indicators over long periods, dynamically adjust diagnostic thresholds, continuously train diagnostic models, thereby reducing misdiagnosis and missed diagnoses. Combined with some mobile applications, AI can synchronize patient conditions with their doctors, allowing users to receive timely medical advice and achieve remote personalized medical care. However, despite these prospects, the actual clinical application of AI-driven nanosensors remains limited. To accelerate clinical translations, standardization, benchmarking, and multicenter validation are crucial. Establishing standardized SOPs, performance metrics, and deidentified datasets can support reproducible evaluation and regulatory approval. Meanwhile, clinical applications require robust engineering and comprehensive product lifecycle management. Nanosensors must demonstrate good consistency and stability, while AI models require regular update and retraining procedures. Together, these can bridge the gap between proof-of-concept studies and real-world clinical applications.

Beyond technological improvement, a fourth important direction of future research involves balancing privacy and the progress of AI-assisted healthcare. Approaches such as federated learning can train AI model locally, thereby avoiding the need to share patient data across institutions and enhancing the data privacy [132]. Edge computing is also an effective way to enhance data privacy and security by processing data locally or near the source rather than relying solely on remote cloud servers. In addition, this method helps to reduce latency and network congestion, lower dependence on continuous connectivity, and improve overall system reliability [133]. Additional strategies, including encryption, differential privacy, and mixed-model approaches, are also expected to strengthen patient confidentiality within AI healthcare systems [134]. Furthermore, the establishment of effective and mature legal and regulatory frameworks, both at the national level and in alignment with World Health Organization guidelines, will be essential to achieve adequate oversight and better transparency [135]. 

Overall, the future development of AI-assisted nanosensors for disease diagnostics is expected to evolve from single disease detection toward multimodal integrated diagnostic models, followed by personalized real-time monitoring, predictive and interventional applications, and ultimately clinically interpretable and implementable systems, while being deeply integrated with emerging fields such as synthetic biology.

## 6. Conclusions

Nanosensors offer notable advantages such as high sensitivity, great specificity, and rapid response, and they have been widely used for the early and accurate diagnosis of various diseases. With the rapid development of clinical medicine, the demand for greater diagnostic performance is increasing, requiring nanosensors with enhanced performance and more sophisticated data analysis capabilities. The integration of AI has brought new power to the development of clinical diagnostic sensors, enabling fast sensor design, optimization, and predictive modeling, while facilitating precise analysis of complex, diverse, and large-scale datasets. AI technologies have greatly expanded the potential of nanosensors in disease diagnostics. This review has outlined the recent advances on AI-assisted nanosensors across diverse diagnostic scenarios, including their incorporation into wearable devices and IoT-based healthcare systems, while also highlighting challenges and prospective directions. In the context of precision and personalized medicine, AI-assisted nanosensors are transformative. Applying appropriate models to different sensors and data types, AI has huge potential to improve diagnostic nanosensors, thereby contributing to the early detection and effective management of diseases that threaten global health.

## Figures and Tables

**Table 1:** AI-assisted nanosensors utilized in clinical diagnostics and their performance metrics.

Disease	Target Analyte	Detection Device	AI Algorithm	Performances	Ref.
COVID-19	Protein/virus RNA/antigen/Ab/blood pressure	Various sensors	SSO-GDCFN	Accuracy: 99.99%; precision: 99.98%; sensitivity: 100%; specificity: 95%; kappa: 0.965; AUC: 0.98	[71]
COVID-19	Spike protein’s RBD	Bio-inspired peptide based electrochemical sensor	SVM	Accuracy: 90%; sensitivity: 100%; specificity: 80%	[73]
COVID-19	Virus RNA	Chemiresistive biochip	SVC/RBF	Accuracy: 95.2% and 100% (for extracted and non-extracted RNA samples)	[74]
Sexually transmitted diseases	Sexually transmitted viruses	2D photonic crystal-based optical sensor	KNN	Accuracy: 97.12%; sensitivity: 998 nm; quality factors: 3988	[75]
COVID-19	Nucleoprotein	SERS sensor	DL	Accuracy: 94.52% and 100% (for testing and training set)	[76]
COVID-19	S protein, N protein, VLP protein, Streptavidin	SERS sensor	DeepATsers (based on CNN and GAN)	Accuracy: 97.5%	[77]
Dengue fever	Protein	Optoelectronic biosensor	ANN (trained by Salp swarm algorithm)	-	[78]
Malaria	Malaria parasite	Tunable terahertz metasurface biosensor	ML (XGBoost)	Sensitivity: 429 GHzRIU^−1^; detection accuracy: 25.6; a figure of merit: 10.989 RIU^−1^; R^2^ > 99%	[60]
Tuberculosis	*Mycobacterium tuberculosis*	Graphene-based terahertz metamaterial biosensor	ML (XGBoost)	Sensitivity: 1000 GHzRIU^−1^; accuracy: 100%; R^2^: 98.825%	[79]
COVID-19	Six SARS-CoV-2 antigens	Nanoplasmonic biosensor	RF	-	[80]
Zika virus disease	Zika virus	MIP-based electrochemical sensor	SVM	Accuracy: 91%	[81]
Breast cancer	miRNA-21, miRNA-96	Lateral flow chip sensor	SVR, GPR	Detection time: <5 min; LOD: 10^−15^ M level; R^2^: 99.16%	[82]
Cancers	Cancer cells	2D-photonic crystal biosensor	ANN	Sensitivity: 55%-89%; highest accuracy: 81%	[83]
Prostate cancer	Exosomal TMEM256, flotillin-2, PSMA	DGFET biosensor	XAI	Accuracy: 66.7% (PI-RADS 3); AUC: 0.93	[84]
Cancers	HeLa, PC12, MDA, MCF, and Jurkat cells	2D photonic crystal biosensor	MLR, SVM	R^2^: 99% (SVM), 88% (MLR)	[85]
Cervical cancer	Cervical cancer tissues	Metasurface SPR sensor	SVR with polynomial kernel	Sensitivities: 400 GHzRIU^−1^; figures of merit: 5.882 RIU^−1^; quality factors: 9.206-9.950; R^2^: 100%	[86]
Oral cancer	Cystatin B, leukotriene A 4 hydrolase, and collagen type VI alpha 1 chain in human saliva	Electrochemical sensor	ML-based AdaBoost model	LOD: 0.4 ng/mL; accuracy: 76%	[87]
Cancer	CEA	Split-ring resonator microwave biosensor	CNN	LOD: 39 pg/mL; sensitivity: 27.5 MHz/(ng/mL); R^2^: 99.9%	[88]
Breast cancer	ECM1	Electrochemical immunosensor	ANN	Linear range: (0.1-7.5) ×10^−9^ M; LOD: 1.2×10^−11^ M; current retention over 1 h: 98.6%	[89]
Cancer	p53	Electrochemical immunosensor	ML	-	[90]
Breast cancer	miRNA-451, miRNA-145	Bipolar self-powered electrical sensor	RF	Linear range: 10^−15^–10^−10^ M; LOD: 2.4 × 10^−16^ M and 3.17 × 10^−16^ M	[91]
Lung cancer	Salivary biomarkers	3D-printed microfluidic SERS sensor	SVM	Accuracy: 94%; sensitivity: 93.5%; specificity: 88%	[92]
Cardiovascular disease	-	Medical sensors and wearable devices	Shuffled Frog leaping-tuned iterative improved adaptive boosting algorithm	Accuracy: 95.37%; precision: 93.51%; sensitivity: 94.3%; specificity: 96.31%; F-measure: 95.72%	[93]
Cardiovascular disease	-	Various sensors	Bidirectional-gated recurrent unit attention model	Accuracy: 99.90%	[94]
Cardiovascular disease	Blood pressure, ECG, heart rate	Wearable sensors	ML with stacking classifier	F1 score: 80.4% and 91% (3-level and 2-level system)	[95]
Cardiovascular disease	-	Various sensors and sources	ML and fuzzy logic	Accuracy: 82.6%; precision: 81.5%; recall: 83.7%; F1 score: 82.5%	[96]
Cardiovascular disease	Cholesterol	SERS sensor	RF	Accuracy: 83.5%; LOD: 10^−8^ M	[97]
Cardiovascular disease	-	Wearable ECG and bioimpedance device	ML with LightGBM classifier	Accuracy: 94.49%	[98]
Metabolic disorders	Dimethylamine, creatinine, glucose, and H^+^	MIP-based wearable sensor	MLP	Accuracy: >98%	[99]
Diabetes	Glucose, lactate	Electrochemiluminescence biosensor	ML	LOD: 1.42 × 10^−4^ M (glucose), 3.42 × 10^−4^ M (lactate)	[100]
Diabetes	Glucose	Photosensitive sensor	Federated learning	Root mean square error: 19.1 mg/dL	[101]
Diabetes	ECG	Wearable sensor	Edge-AI	Accuracy: 89.52%	[102]
Diabetes	Sweat pH and glucose	Wearable sensor	RF, CNN	R^2^: 99%	[103]
Neurodegenerative diseases	Proteins	Immuno-nano-plasmonic infrared metasurface sensor	DNN	Accuracy: 94.66%	[104]
Alzheimer’s disease	Amyloid β (1–42) and metabolites	SERS sensor	DL	Accuracy: 96–100%	[105]
Alzheimer’s disease	Aβ40/Aβ42 aggregates	Fluorescent sensor	ML	Accuracy: 100%	[106]
Alzheimer’s disease	Aβ40, Aβ42, P-tau181, P-tau217, NfL	Graphene FET sensor	ML	AUC: 0.94	[107]

## Data Availability

No new data were created or analyzed in this study.

## Abbreviations

The following abbreviations are used in this manuscript:

Abs	Antibodies
AgNPs	Silver nanoparticles
AI	Artificial intelligence
AIDS	Acquired immunodeficiency syndrome
ANN	Artificial neural network
AUC	Area under the curve
CNN	Convolutional neural network
CTCs	Circulating tumor cells
DGFET	Dual-gate field-effect transistor
DL	Deep learning
DNN	Deep neural network
ECM1	Extracellular matrix protein-1
ELISA	Enzyme-linked immunosorbent assay
FDT	Fuzzy-based decision tree
FET	Field-effect transistor
FFNN	Feedforward neural networks
FNDs	Fluorescent nanodiamonds
GANs	Generative adversarial networks
GDCFN	Generalized deep convolutional fuzzy network
GNPs	Gold nanoparticles
GO	Graphene oxide
GPLMs	General protein language models
GPR	Gaussian process regression
IoT	Internet of Things
KNN	k-Nearest Neighbors
LMs	Language models
LOD	Limit of detection
LSTM	Long short-term memory
MERS-CoV	Middle East respiratory syndrome coronavirus
MIPs	Molecularly imprinted polymers
ML	Machine learning
MLP	Multilayer perceptron
MLR	Multiple linear regression
MS	Mass spectrometry
MWCNTs	Multi-walled carbon nanotubes
NMs	Nanomaterials
PCR	Polymerase chain reaction
PI-RADS	Prostate imaging reporting and data system
POCT	Point-of-care testing
PSO	Particle swarm optimization
RBD	Receptor binding domain
RBMs	Restricted Boltzmann machines
RF	Random forest
RNN	Recurrent neural network
SARS-CoV	Severe acute respiratory coronavirus
SELEX	Systematic evolution of ligands by exponential enrichment
SERS	Surface-enhanced Raman scattering
SPR	Surface plasmon resonance
SSO	Shuffle shepherd optimization
SVC/RBF	Support vector classifier with a radial basis function
SVM	Support vector machine
SVR	Support vector regression
VAEs	Variational autoencoders
VOC	Volatile organic compound
XAI	Explainable artificial intelligence
